# Epigenetic Liquid Biopsy Marks Atrial Fibrillation: Evidence from the AF Big Picture Study

**DOI:** 10.3390/epigenomes10010009

**Published:** 2026-02-05

**Authors:** Riccardo Proietti, Nicola Tidbury, Joshua Preston, Maanya Vittal, Philippa McCabe, Garry McDowell, Gregory Y. H. Lip, Manlio Vinciguerra

**Affiliations:** 1 Liverpool Centre for Cardiovascular Science at the University of Liverpool, Liverpool John Moores University and Liverpool Heart and Chest Hospital (LHCH), Liverpool L14 3PE, UK; riccardo.proietti@liverpool.ac.uk (R.P.); nicola.tidbury@lhch.nhs.uk (N.T.); joshua.preston@lhch.nhs.uk (J.P.); g.mcdowell@ljmu.ac.uk (G.M.); gregory.lip@liverpool.ac.uk (G.Y.H.L.); 2 Department of Cardiovascular and Metabolic Medicine, Institute of Life Course and Medical Sciences, Faculty of Health and Life Sciences, University of Liverpool, Liverpool L69 7ZX, UK; 3 School of Pharmacy and Biomolecular Science, Faculty of Science, Liverpool John Moores University, Liverpool L3 3AF, UK; m.vittal@ljmu.ac.uk (M.V.); p.g.mccabe@ljmu.ac.uk (P.M.); 4 Danish Center for Health Services Research, Department of Clinical Medicine, Aalborg University, 2450 Aalborg, Denmark; 5 Department of Cardiology, Lipidology and Internal Medicine with Intensive Coronary Care Unit, Medical University of Bialystok, 15-274 Bialystok, Poland; 6 Research Institute, Medical University Varna, 9002 Varna, Bulgaria; 7 Department of Medicine and Surgery, LUM University, 70010 Casamassima (BA), Italy

**Keywords:** atrial fibrillation, liquid biopsy, histones, epigenetics, blood

## Abstract

**Background/Objectives**: Atrial fibrillation (AF) is currently the most common arrhythmia worldwide, and it is linked to increased mortality and morbidity, hence the need for a better clinical stratification of AF patients. Histone complexes or nucleosomes, released into the blood circulation, are found elevated in acute conditions such as stroke, trauma, and sepsis. The aim of this pilot single-centre study was to assess whether circulating histone levels could be used for diagnostic purposes in patients with AF. **Methods**: A total of 40 patients, well characterised for their biochemical and clinical characteristics, were recruited from outpatient clinics. Patients were randomly recruited into two groups (n = 20 per group), i.e., persistent AF and hypertensive controls. A multi-channel flow imaging methodology based on ImageStreamX was used with a well-optimised protocol to image and quantify five individual histones (H2A, H2B, H3, H4, and macroH2A1.1) together with the dimers (H2A/H2B, and H3/H4). **Results**: In the AF groups, plasma levels of histone dimers H2A/H2B and H3/H4 were elevated compared to hypertensive controls, 1.8% vs. 1.06% (*p*-value = 0.03). H2A/H2B dimer levels were increased in AF patients irrespective of gender, smoking status, diabetes, and pharmacological therapy. In the overall population, an inverse correlation between H2A and BMI was detected. **Conclusions**: Our pilot study, although limited in sample size, suggests that circulating histone complexes may be epigenetic sentinels for AF, offering mechanistic insights while addressing unmet needs in risk stratification.

## 1. Introduction

Atrial fibrillation (AF) is the most common arrhythmia worldwide, and a steady rise is forecast, driven by increased life expectancy in the general population and a growing focus on identifying undiagnosed AF [1]. It is projected that the number of patients with AF will double over the next 40 years [2].

AF is a recognised risk factor for increased mortality and morbidity from stroke, dementia, and heart failure. Indeed, AF is associated with a 3–5-fold higher risk of stroke, with the risk associated with several comorbidities [3]. The need for a better clinical stratification of patients with AF and the link of duration of arrhythmias with biological changes that characterise the clinical prognosis has paved the way to the investigation of surrogate biomarkers to implement in clinical practice.

Blood biomarkers of inflammatory and coagulation activity as well as myocardial injury have been tested in selected cohorts of patients with AF [4,5]. Though plausible pathophysiological mechanisms may underline the use of myocardial necrosis and inflammation and coagulation biomarkers with the burden of AF, their clinical usefulness in risk stratification of patients with AF and their use to potentiate the discriminatory power of current clinical scores remain marginal. More recently, miRNA has been put forward as a biomarker with high sensitivity and specificity to identify patients at risk of developing AF, as they exhibit stable expression post-sample collection and can reflect the complex pathological processes like fibrosis and inflammation that existing clinical markers like BNP or CHA2DS2-VASc scores do not fully capture [6,7].

Chromatin is the complex of histones and DNA in the cell nucleus. Its basic repeating unit, the nucleosome, is composed of 146 base pairs of DNA wrapped around an octameric core of histones (H2A, H2B, H3, H4). Histone complexes or nucleosomes, released into the blood circulation, are elevated in several types of cancer and in acute conditions such as stroke, trauma, and sepsis [8,9]. Histones have a long half-life and are very stable in the blood.

We have recently shown that plasma-circulating histones and histone complexes help diagnose non-alcoholic fatty liver disease in children and adults [10,11,12,13]. Also, we recently found increased levels of most of the histones and histone complexes in both HF with preserved ejection fraction (HFpEF) and HF with reduced ejection fraction (HFrEF) patients, and histone H2A was significantly elevated only in HFpEF, compared to healthy individuals [14]. In addition, extracellular histones are key components of neutrophil extracellular traps (NETs), whose formation is enhanced in AF [15].

In this single-centre study, the ‘AF Big Picture Project’, recruiting at Liverpool Heart and Chest Hospital NHS Foundation Trust and using established and emerging biomarkers, we aimed to investigate the pathological mechanisms of AF development and progression with a cross-sectional design, examining the differences between patients with no documented AF but who are diagnosed with hypertension and those with paroxysmal and persistent AF. The focus of this pilot study was to assess whether circulating histone levels could be used in AF for diagnostic purposes.

## 2. Results

### 2.1. Patients

We enrolled 40 patients: 20 with persistent atrial fibrillation (AF) and 20 hypertensive controls with no documented atrial fibrillation (non-AF). The AF group was significantly older compared to the control group (Table 1). There was no difference in BMI, mean systolic and diastolic blood pressure, and prevalence of comorbidities and risk factors, including prior cardiovascular events and smoking between the two groups (Table 1). Patients with AF were more commonly treated with beta-blockers compared to the hypertensive controls. Additionally, 90% of the AF patients were on anticoagulation therapy, whereas none of the hypertensive controls were anticoagulated.

### 2.2. Levels of Circulating Histones in AF Versus Non-AF

**Figure 1:**
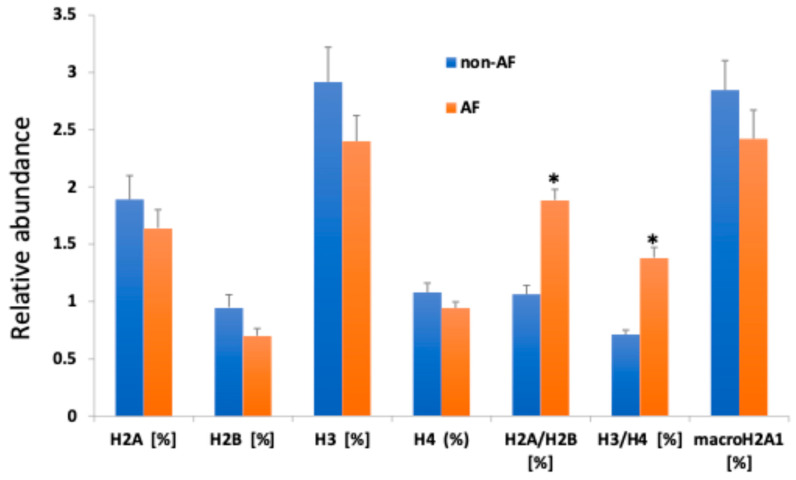
The relative abundance of circulating histones and histone complexes (expressed as relative abundance) as measured by ImageStreamX in the plasma of patients with (n = 20) or without AF (n = 20). The results are expressed as the average plus minus SEM. Significance is indicated by asterisks: <0.05 (*) versus non-AF, based on the Kruskal–Wallis test, followed by a post-hoc test.

Five individual histones (H2A, H2B, H3, H4, and macroH2A1.1) together with the dimers (H2A/H2B, and H3/H4) were assayed by ImageStreamX, i.e., imaging flow cytometry. In the AF groups, plasma levels of histone dimers H2A/H2B and H3/H4 were elevated compared with hypertensive controls (non-AF), 1.8% vs. 1.06% (*p*-value = 0.03) and 1.3% vs. 0.71% (*p*-value = 0.04), respectively (Figure 1).

**Figure 2:**
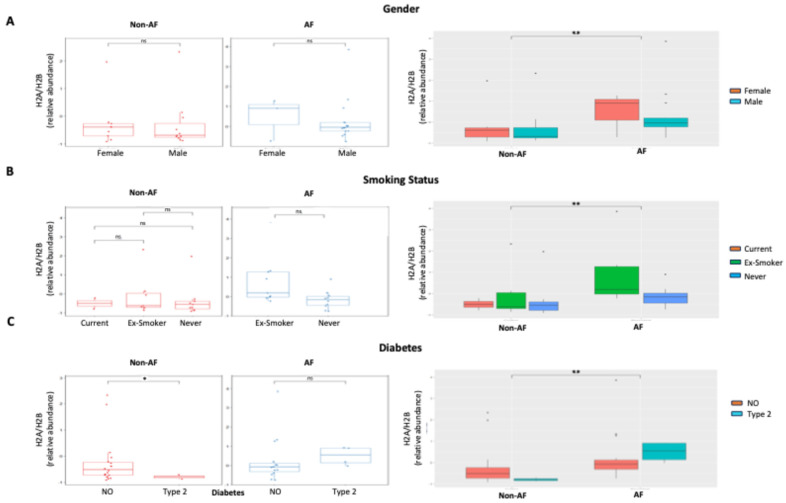
Differences in H2A/H2B histone dimer levels between patients with (n = 20) or without (n = 20) persistent AF, according to gender (**A**), smoking status (current, ex-smoker, never) (**B**), or type 2 diabetes (**C**). Significance is indicated by asterisks: <0.01 (**), <0.05 (*) based on the Mann–Whitney test. ns = non-significant.

**Figure 3:**
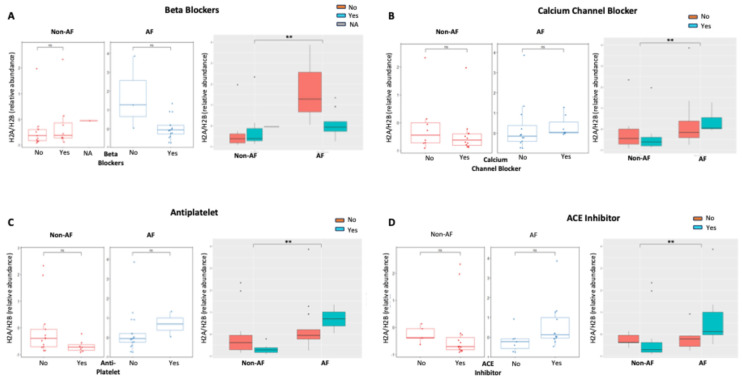
Differences in H2A/H2B histone dimer levels between patients with (n = 20) or without (n = 20) AF, according to the administration of beta-blockers (**A**), calcium channel blockers (**B**), antiplatelets (**C**), or ACE inhibitors (**D**). Significance is indicated by asterisks: <0.01 (**) based on the Mann–Whitney test. ns = non-significant.

Of note, individual histone levels were not significantly different between the two groups. Using a methodology different from imaging flow cytometry, commercial enzyme-linked immunosorbent assay (ELISA), individual H2A, H2B, H3, and H4 were measured in patient plasma. Histone levels were well below the calibration range (3.13–200 ng/mL) and did not differ between the two groups (Figure S1). H2B was not detectable in either group. In addition, H2A/H2B dimer levels were significantly increased in AF patients irrespective of gender, smoking status, diabetes, and pharmacological therapy (Figure 2A–C, Figure 3A–D; right panels, grey filled).

**Figure 4:**
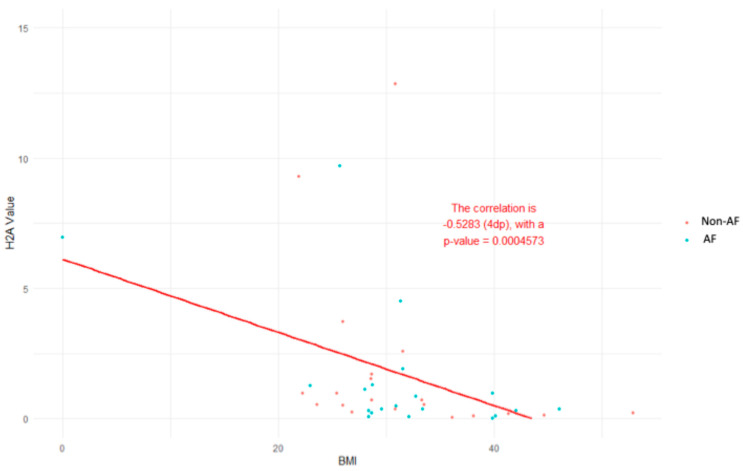
The correlation between H2A levels (arbitrary units) and BMI in the overall population, composed of 20 patients without persistent AF (in red) and 20 patients with persistent AF (in blue). A nonparametric Spearman correlation was applied.

Within each group (non-AF and AF), neither gender nor smoking nor pharmacological therapy were associated with significant differences in H2A/H2B (Figure 2A,B, Figure 3A–D, left panels, white filled), with the exception of a significant decrease in H2A/H2B levels in diabetic patients in the non-AF group (Figure 2C, left panel). In the subgroup analysis carried out according to the same clinical variables, there was no difference in the plasma levels of the H3/H4 (Figures S2–S7). In the overall population, a moderate inverse correlation between H2A and BMI was detected (Figure 4, Table 2), while none of the histone species levels correlated with any of the other clinical variables (age, systolic and diastolic blood pressure, heart rate) (Table 2).

## 3. Discussion

The main findings of this single-centre pilot study can be summarised as follows: (i) histone dimers H2A/H2B and H3/H4 are elevated in the plasma of patients with persistent AF compared to hypertensive controls; (ii) the levels of histone dimers H2A/H2B but not H3/H4 were increased in patients with AF independently from clinical variables and pharmacological therapy; (iii) in the overall population, an inverse significant correlation was observed only for the H2 monomer and BMI.

AF represents a major global health challenge, with its prevalence projected to double over the next four decades due to ageing populations and improved detection strategies [16]. Current clinical risk stratification tools effectively guide anticoagulation decisions but fall short in capturing the underlying biological heterogeneity of AF, particularly the progressive atrial remodelling and inflammatory burden that distinguish paroxysmal from persistent forms.

In this study, we explored circulating histone complexes as a novel epigenetic liquid biopsy for AF, leveraging their stability in plasma and established role as damage-associated molecular patterns (DAMPs) in cardiovascular pathologies [17]. Our findings demonstrate distinct alterations in the relative abundances of histone monomers (H2A, H2B, H3, H4) and complexes (H2A/H2B, H3/H4, macroH2A1) in patients with AF compared to non-AF controls, with significant elevations in H3/H4 and macroH2A1 in the AF cohort (Figure 1). Moreover, our data support the notion that imaging flow cytometry is superior to ELISA for circulating plasma histone detection in terms of sensitivity. These changes were independent of key confounders such as gender, smoking status, and diabetes (Figure 2) and were not substantially influenced by common AF pharmacotherapies, including beta-blockers, calcium channel blockers, antiplatelets, and ACE inhibitors (Figure 3). Furthermore, H2A levels exhibited a negative correlation with body mass index (BMI) across the cohort (r = −0.528, *p* = 0.00047; Figure 4), underscoring a potential interplay between metabolic factors and epigenetic markers in AF progression [16,18].

The observed dysregulation of circulating histones might align with emerging evidence implicating chromatin remodelling in AF pathogenesis [19,20]. Histones, as core components of nucleosomes, are released into circulation during cellular stress, apoptosis, or NETosis—processes amplified in AF due to atrial stretch, oxidative stress, and thromboinflammation. Specifically, the elevated H3/H4 complex in AF patients may reflect the possibility of enhanced nucleosome eviction and transcriptional activation of pro-fibrotic and pro-inflammatory genes in atrial cardiomyocytes. Interestingly, macroH2A1, a variant histone associated with gene silencing and metabolic regulation, has been linked to hepatic steatosis and insulin resistance [11,18,21,22,23,24,25]; however, its levels in AF plasma remain unchanged. Notably, the lack of significant differences in individual monomers (H2A, H2B, H3, H4) but prominence in complexes suggests that oligomeric forms are more sensitive biomarkers, possibly due to their resistance to plasma degradation and higher pathophysiological relevance as signalling entities.

About 30% of patients in our study, in either the non-AF or AF group, experienced past cardiovascular events. During a myocardial infarction in animal models, dying cardiomyocytes sharply release histones, which can be cytotoxic at the beginning of reperfusion [26]. However, within 1–2 days, histone levels return to basal pre-event levels [26]. Therefore, it is unlikely that past cardiovascular events would represent a confounding factor for the measurement of plasma histone levels in our patients.

These histone profiles were robust across demographic and clinical subgroups, highlighting their potential as a universal AF marker. Gender-stratified analyses revealed no sex-specific biases (Figure 2A). Smoking status and diabetes did not confound histone abundances (Figure 2B,C), implying that these epigenetic signals capture AF-specific biology beyond traditional risk factors. Medication effects were minimal (Figure 3), with only modest trends in beta-blocker users potentially attributable to heart-rate modulation influencing nucleosome turnover rather than direct histone interference. The moderate inverse H2A-BMI relationship (Figure 4) is intriguing, as obesity is a potent AF trigger via adipokine-mediated inflammation; lower H2A in higher BMI may indicate histone sequestration in adipose tissue or altered chromatin accessibility in obese atrial tissue, warranting mechanistic studies. Of note, the non-AF control group consisted of hypertensive subjects who were a few years younger than the AF group. Age is a well-known determinant of AF, and we cannot exclude that it could play a confounding effect due to the potential higher baseline levels of predictive biomarkers (NT-proBNP, inflammatory cytokines, etc) and/or and more advanced structural/functional atrial changes.

Clinically, this epigenetic liquid biopsy, if confirmed in larger cohorts, offers transformative potential for AF management. Unlike volatile miRNAs or transient troponins, histones’ long half-life and plasma stability enable point-of-care detection, facilitating early screening in at-risk populations (e.g., post-stroke or hypertensive cohorts) and monitoring treatment response in persistent AF [6,27,28]. Integrating histone panels into existing scores could enhance predictive accuracy for thromboembolism or progression to persistent disease, where current biomarkers, such as NT-proBNP, show limited specificity. As non-invasive alternatives to invasive biopsies, these markers could guide personalised therapies, such as targeting HDAC inhibitors to reverse AF-associated chromatin changes [29,30]. Patients with persistent AF face heightened risks of stroke and mortality compared to paroxysmal forms, underscoring the need for such advanced stratification [13,31].

Our study has limitations. The cross-sectional design precludes causal inferences; longitudinal validation in incident AF cases is essential to track histone dynamics over arrhythmia burden. Sample size, while powered for primary endpoints, is limited and may limit subgroup analyses (e.g., paroxysmal vs. persistent), and ethnic diversity was not fully represented, potentially affecting generalizability. Assay standardisation remains a hurdle, as flow cytometry-based detection requires optimisation for high-throughput clinical use. Future multicentre trials should incorporate functional assays and epigenome-wide association studies to dissect AF-specific modifications like methylation or acetylation in larger samples. In conclusion, circulating histone complexes emerge as promising epigenetic sentinels for AF, offering mechanistic insights into its inflammatory and remodelling axes while addressing unmet needs in risk stratification. By harnessing this liquid biopsy, we move closer to precision cardiology, where molecular fingerprints inform proactive care and mitigate the escalating burden of AF.

## 4. Materials and Methods

### 4.1. Patients and Biofluids

A total of 40 patients were recruited from the outpatient clinic at Liverpool Heart and Chest Hospital NHS Foundation Trust (IRAS: 265408. REC: 21/EE/0040). Patients were recruited into two groups (n = 20 per group): persistent AF and hypertensive controls. Persistent AF patients were defined as any patient in AF for greater than 7 days and not self-terminating, and hypertensive controls had no prior diagnosis of AF and had blood pressure of >140/90 mmHg on more than one occasion. As per the study protocol, patients were excluded from the study if they had an inactive or recent malignancy (<3 months), a recent acute coronary syndrome (ACS) event (<6 weeks), active inflammatory or autoimmune disease, or haemophilia. All eligible patients provided written informed consent prior to study participation, demographic data were collected, and blood samples were drawn. Blood samples were spun at 1800× *g* for 10 min, within 2 h of collection. The resulting plasma was aliquoted and frozen at −80 °C until analysis.

### 4.2. ImageStream(X)-Based Detection of Histones and Histone Complexes

A multi-channel flow imaging methodology based on ImageStreamX was used with a previously optimised protocol to image single histone particles on different channels [10,11,14,32,33].

### 4.3. ELISA-Based Detection of Histones and Histone Complexes

To quantify histones H2A, H2B, H3, and H4 levels in blood samples, we employed the Histone ELISA Kit (Catalogue No: abx156690, abx156691, abx153542—abbexa and LS-F5238, LS Bio, Newark, CA, USA). The assay relies on a sandwich enzyme-linked immunosorbent assay (ELISA) technique, which utilises a specific histone-specific antibody pre-coated onto a 96-well microplate to capture H2A, H2B, H3, and H4 in the samples individually. Blood samples were collected and processed immediately to prevent protein degradation. They were aliquoted and stored at −20 °C to −80 °C, avoiding multiple freeze–thaw cycles. On the day of the assay, all samples were brought to room temperature before the experiment. ELISA was carried out in accordance with the manufacturer’s instructions. The optical density (OD) was immediately measured at 450 nm using a microplate reader (Tecan Infinite^®^ M200 Pro (Grödig, Austria)). Therefore, to determine histone concentrations, the average OD values for each standard, sample, and control were calculated. The relative OD was obtained by subtracting the average OD of the control wells from the OD of each sample well. A standard curve was generated by plotting the relative OD values of the standards against their known concentrations. The histones in the patient samples were then interpolated from the standard curve and multiplied by the dilution factor.

### 4.4. Statistical Analyses

Statistical analyses were conducted in Python using the SciPy library (version 1.11.3) and in GraphPad Prism (version 10.2.3). Levels of histones and histone complexes were measured as percentage from the gated object population. Since histones H2B, H3, and H4 were measured in all three sets of antibodies, the abundance of single histones and histone complexes was log-transformed, scaled, and averaged to account for potential batch effect. To compare the histone abundances between the two groups, we applied the Kruskal–Wallis test, followed by a post-hoc test. Whisker boxplots have been generated through matplotlib (version 3.8.1) and GraphPad Prism (version 10.2.3). To correlate histone levels with the indicated parameters (Table 2), we used nonparametric Spearman’s correlation and Bonferroni multiple testing correction method. To compare histone levels with the indicated parameters within the respective patient groups stratified by sex, we applied the Mann–Whitney test. Significant differences were indicated by asterisks: <0.05 (*), <0.01 (**), and <0.001 (***).

## Figures and Tables

**Table 1:** Patient data. ( ) = standard deviation. * = significant as of Mann–Whitney U test.

	AF (n = 20)	Non-AF (n = 20)	*p* Value
Age (years)	71 (14.75)	60.5 (18.0)	0.0239 *
BMI	31.44 (6.43)	29.73 (8.18)	0.3945
Gender (% male)	80	55	0.176
Systolic blood pressure (mm Hg)	126.5 (30.0)	137.5 (23.0)	0.1125
Diastolic blood pressure (mm Hg)	79.5 (14.25)	83.5 (16.25)	0.3366
Heart Rate (bpm)	81.5 (14.25)	76.5 (9.5)	0.0682
Smoking status (%):			
Never	55	55	
Ex-smoker	45	35	0.325
Current	0	10	
Diabetes (%)	20	10	0.6614
Thyroid disease (%)	10	5	>0.9999
Previous Cardiovascular Event (%)	30	30	>0.9999
Medications (%):			
Beta-blocker	85	45	0.0187
Anti-arrhythmic	15	0	0.2308
Calcium channel blocker	35	60	0.2049
Anticoagulant	90	0	<0.0001 *
Antiplatelet	10	35	0.1274
ACE inhibitor/ARB	60	75	0.5006
Digoxin	5	0	>0.9999

**Table 2:** A list of variables with their correlation and *p*-values, with a significant *p*-value highlighted in red for each of the histone monomers and dimers. Here, *p* = *p*-value and c = correlation value.

	Age	BMI	Systolic Blood Pressure	Diastolic Blood Pressure	Heart Rate
**H2A**	*p* = 0.8682 c = 0.0271	*p* = 0.00046 c = −0.5283	*p* = 0.0591 c = 0.3011	*p* = 0.8774 c = −0.0252	*p* = 0.3619 c = −0.1481
**H2B**	*p* = 0.3465 c = −0.1528	*p* = 0.6775 c = 0.0678	*p* = 0.4557 c = −0.1213	*p* = 0.5346 c = −0.1011	*p* = 0.5875 c = −0.0884
**H3**	*p* = 0.7655 c = 0.0487	*p* = 0.1522 c = −0.2306	*p* = 0.7168 c = 0.0592	*p* = 0.4832 c = −0.1142	*p* = 0.7811 c = 0.0454
**H4**	*p* = 0.8549 c = 0.0298	*p* = 0.6077 c = 0.0837	*p* = 0.1248 c = 0.2467	*p* = 0.2264 c = 0.1956	*p* = 0.8139 c = −0.0384
**H2A/H2B**	*p* = 0.2341 c = 0.1951	*p* = 0.2063 c = −0.2069	*p* = 0.8707 c = 0.0269	*p* = 0.7823 c = 0.0457	*p* = 0.5774 c = −0.0928
**H3/H4**	*p* = 0.9352 c = 0.0133	*p* = 0.6971 c = 0.0635	*p* = 0.2112 c = 0.2021	*p* = 0.8774 c = 0.0257	*p* = 0.8584 c = 0.0291
**MacroH2A1**	*p* = 0.8977 c = 0.0213	*p* = 0.1178 c = −0.2546	*p* = 0.7192 c = 0.0594	*p* = 0.2359 c = −0.143	*p* = 0.5915 c = −0.0886

## Data Availability

The datasets generated during and/or analysed during the current study are available from the corresponding author on reasonable request.

## Supplementary Materials

The following supporting information can be downloaded at https://www.mdpi.com/article/10.3390/epigenomes10010009/s1; Figure S1. Levels (ng/mL) of individual circulating histones (H2A, H2B, H3, H4) detected by ELISA in the plasma of patients with (n = 20) or without AF (n = 20). Figure S2. Differences in individual histone levels between patients with (n = 20) or without (n = 20) persistent AF according to gender stratification. Significance is indicated by asterisks. ns = non-significant. Figure S3. Differences in individual histone levels between patients with (n = 20) or without (n = 20) persistent AF according to smoking status (current, ex-smoker, never). Significance is indicated by asterisks. ns = non-significant. Figure S4. Differences in individual histone levels between patients with (n = 20) or without (n = 20) persistent AF according to the presence or absence of diabetes. Significance is indicated by asterisks. ns = non-significant. Figure S5. Differences in individual histone levels between patients with (n = 20) or without (n = 20) persistent AF according to the administration of beta-blockers. Significance is indicated by asterisks. ns = non-significant. Figure S6. Differences in individual histone levels between patients with (n = 20) or without (n = 20) persistent AF according to the administration of calcium channel blockers. Significance is indicated by asterisks. ns = non-significant. Figure S7. Differences in individual histone levels between patients with (n = 20) or without (n = 20) persistent AF according to the administration of antiplatelets. Significance is indicated by asterisks. ns = non-significant. Figure S8. Differences in individual histone levels between patients with (n = 20) or without (n = 20) persistent AF according to the administration of ACE inhibitors. Significance is indicated by asterisks. ns = non-significant.
