# Supplementary material for: Epigenetic Liquid Biopsy Marks Atrial Fibrillation: Evidence from the AF Big Picture Study
Source: Epigenomes. 2026 Feb 5;10(1):9. doi: 10.3390/epigenomes10010009 (PMC12922129; doi:10.3390/epigenomes10010009)
Supplement: Supplementary file 1 [file epigenomes-10-00009-s001.zip › Supplemental Figure 8.pptx]

## Slide 1
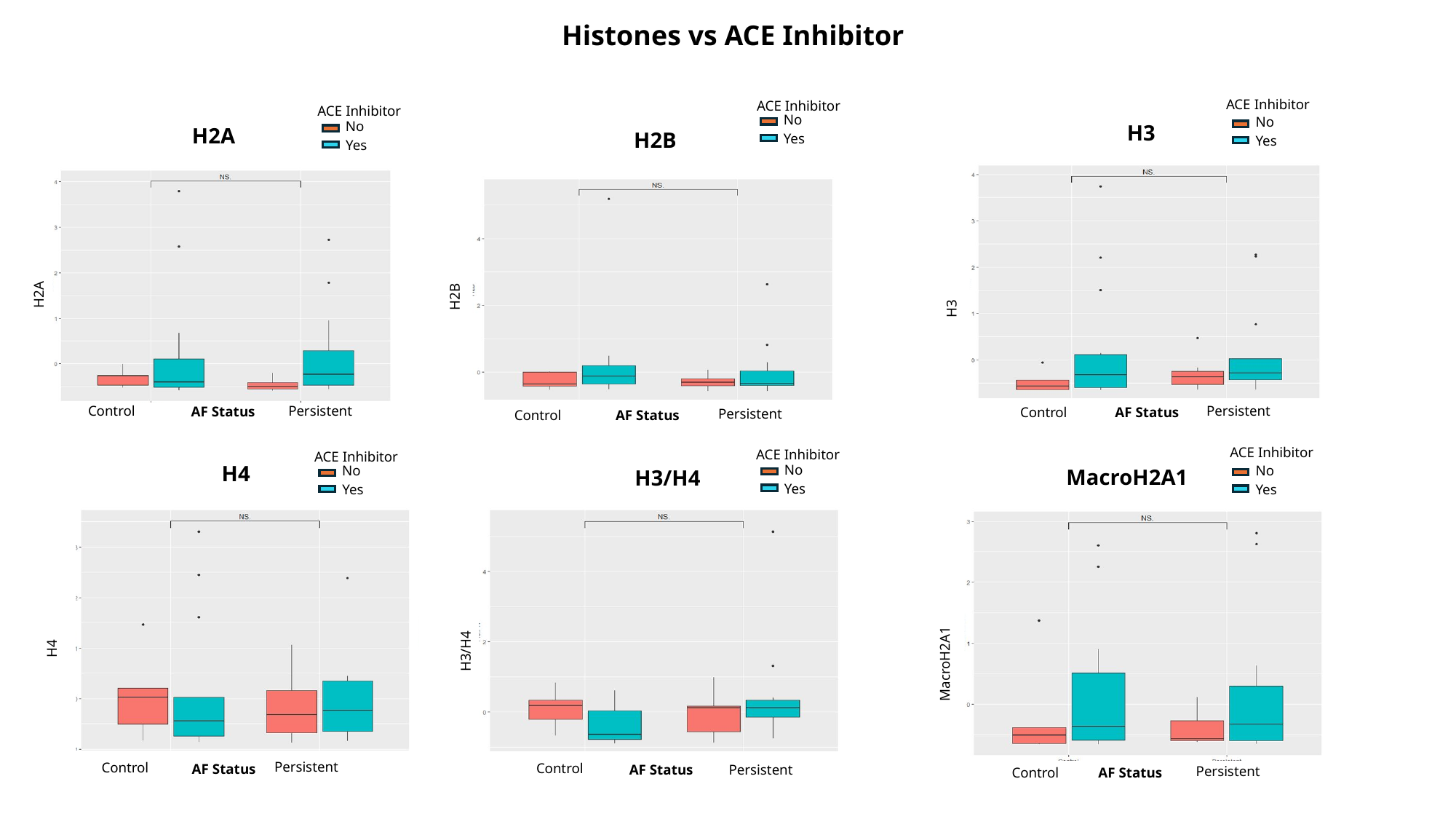

Histones vs ACE Inhibitor
ACE Inhibitor
ACE Inhibitor
ACE Inhibitor
No
No
No
H3
H2A
H2B
Yes
Yes
Yes
H2A
H2B
H3
Control
Persistent
Persistent
AF Status
Control
AF Status
Persistent
Control
AF Status
ACE Inhibitor
ACE Inhibitor
ACE Inhibitor
H4
MacroH2A1
H3/H4
No
No
No
Yes
Yes
Yes
H4
H3/H4
MacroH2A1
Persistent
Control
Control
AF Status
AF Status
Persistent
Persistent
Control
AF Status
