# Supplementary material for: Epigenetic Liquid Biopsy Marks Atrial Fibrillation: Evidence from the AF Big Picture Study
Source: Epigenomes. 2026 Feb 5;10(1):9. doi: 10.3390/epigenomes10010009 (PMC12922129; doi:10.3390/epigenomes10010009)
Supplement: Supplementary file 1 [file epigenomes-10-00009-s001.zip › Supplemental Figure 7.pptx]

## Slide 1
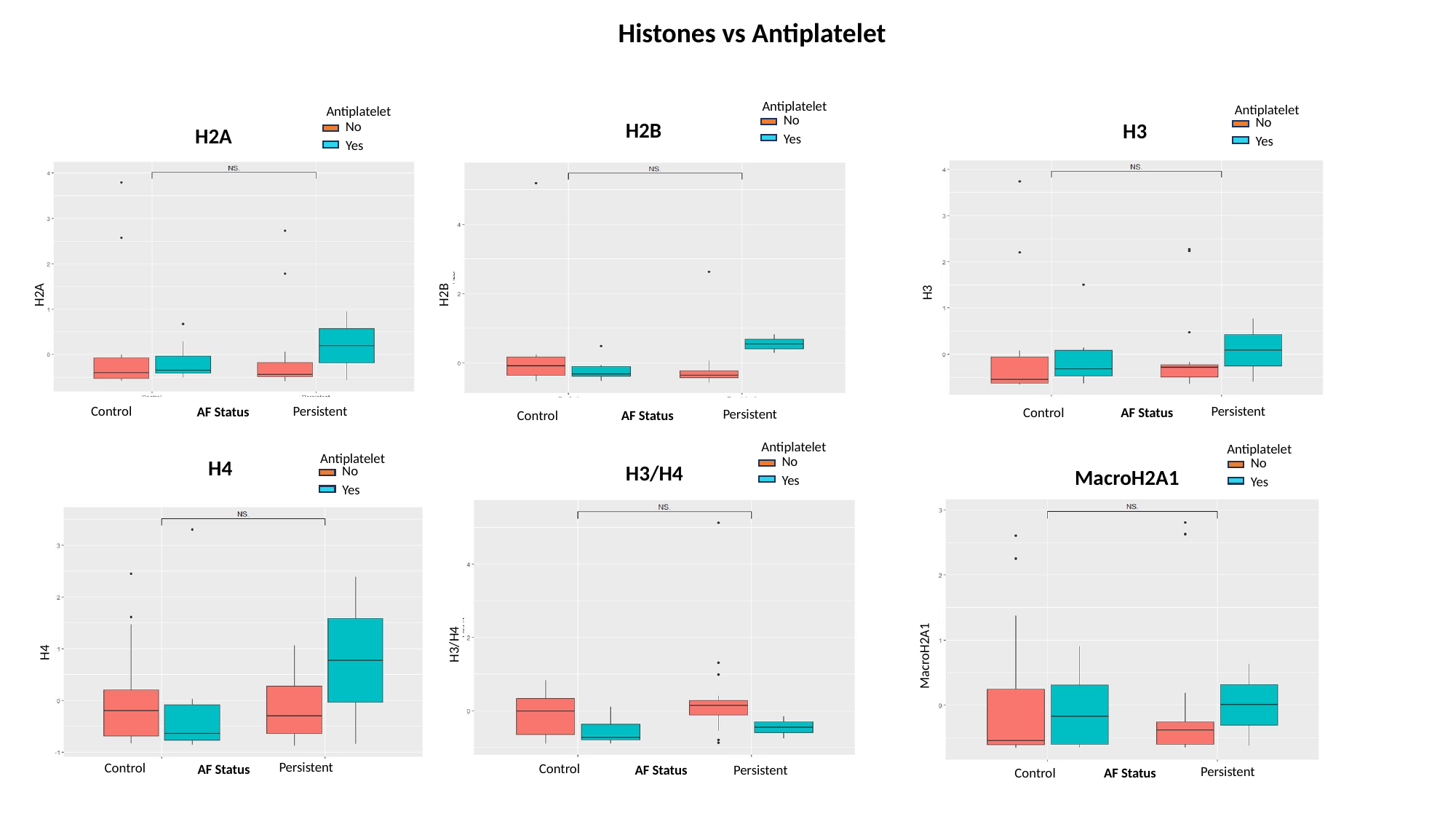

Histones vs Antiplatelet
Antiplatelet
Antiplatelet
Antiplatelet
No
No
H2B
H3
H2A
No
Yes
Yes
Yes
H3
H2B
H2A
Control
Persistent
Persistent
AF Status
Control
AF Status
Persistent
Control
AF Status
Antiplatelet
Antiplatelet
Antiplatelet
No
No
H4
H3/H4
MacroH2A1
No
Yes
Yes
Yes
H3/H4
H4
MacroH2A1
Persistent
Control
Control
AF Status
AF Status
Persistent
Persistent
Control
AF Status
