# Supplementary material for: Epigenetic Liquid Biopsy Marks Atrial Fibrillation: Evidence from the AF Big Picture Study
Source: Epigenomes. 2026 Feb 5;10(1):9. doi: 10.3390/epigenomes10010009 (PMC12922129; doi:10.3390/epigenomes10010009)
Supplement: Supplementary file 1 [file epigenomes-10-00009-s001.zip › Supplemental Figure 3.pptx]

## Slide 1
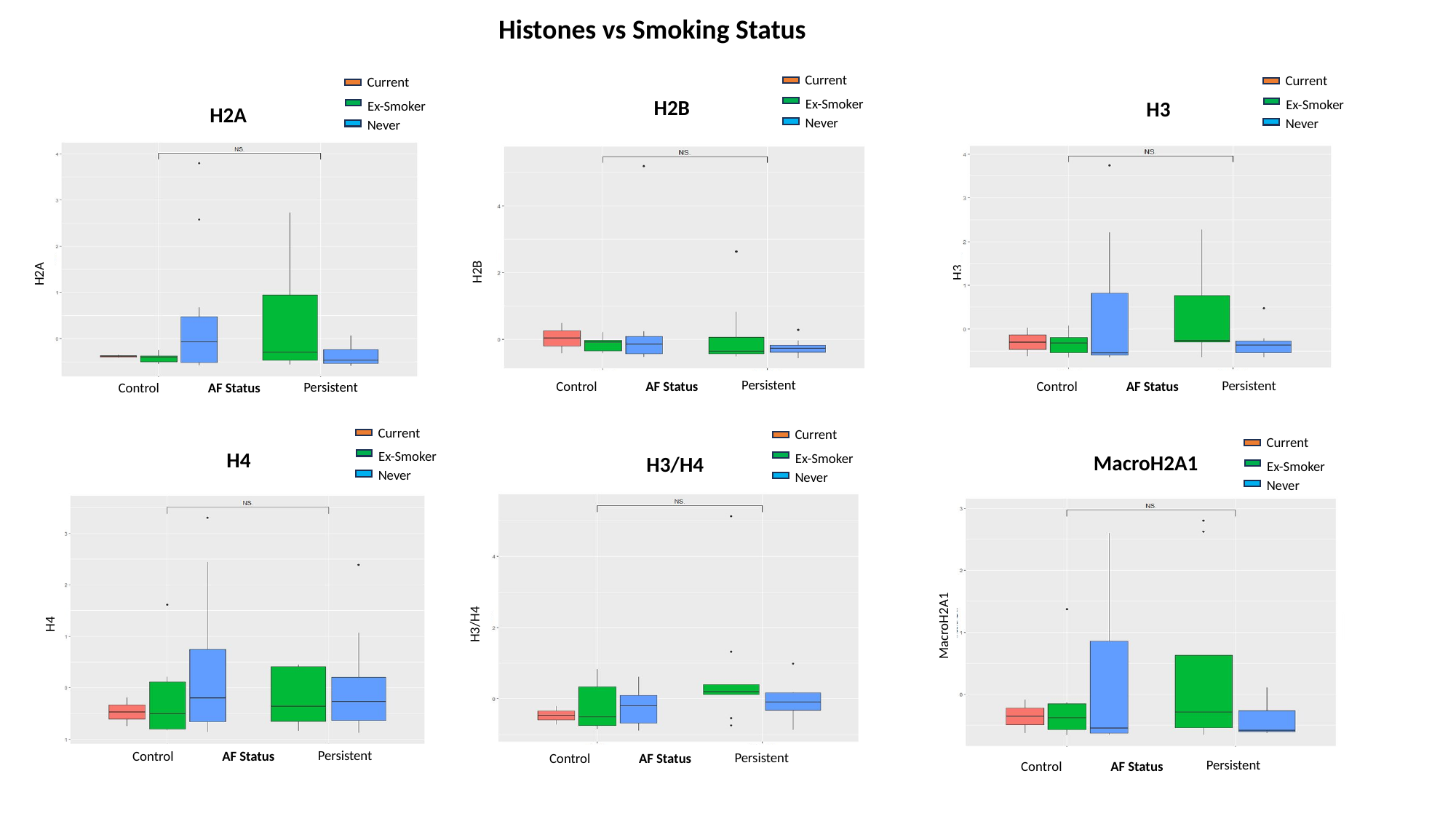

Histones vs Smoking Status
Current
Current
Current
H2B
H3
H2A
Ex-Smoker
Ex-Smoker
Ex-Smoker
Never
Never
Never
H2B
H3
H2A
Persistent
Persistent
Control
AF Status
Control
AF Status
Persistent
Control
AF Status
Current
Current
Current
H4
MacroH2A1
H3/H4
Ex-Smoker
Ex-Smoker
Ex-Smoker
Never
Never
Never
H3/H4
H4
MacroH2A1
Persistent
Control
AF Status
Persistent
Control
AF Status
Persistent
Control
AF Status
