# Supplementary material for: Epigenetic Liquid Biopsy Marks Atrial Fibrillation: Evidence from the AF Big Picture Study
Source: Epigenomes. 2026 Feb 5;10(1):9. doi: 10.3390/epigenomes10010009 (PMC12922129; doi:10.3390/epigenomes10010009)
Supplement: Supplementary file 1 [file epigenomes-10-00009-s001.zip › Supplemental Figure 5.pptx]

## Slide 1
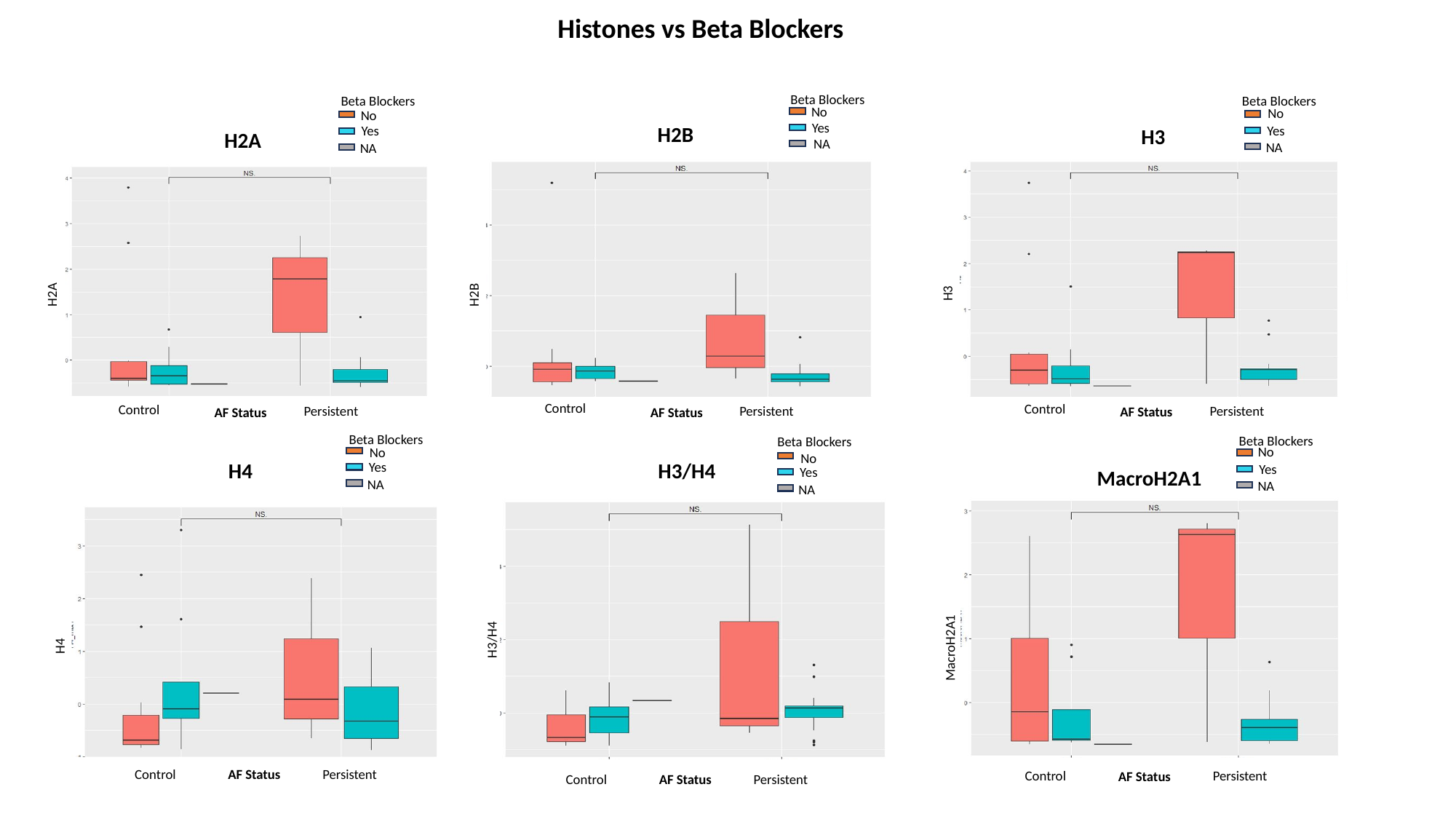

Histones vs Beta Blockers
Beta Blockers
Beta Blockers
Beta Blockers
No
No
No
Yes
H2B
H3
H2A
Yes
Yes
NA
NA
NA
H3
H2A
H2B
Control
Control
Control
Persistent
Persistent
Persistent
AF Status
AF Status
AF Status
Beta Blockers
Beta Blockers
Beta Blockers
No
No
No
H4
H3/H4
MacroH2A1
Yes
Yes
Yes
NA
NA
NA
H3/H4
H4
MacroH2A1
Control
Persistent
AF Status
Control
Persistent
AF Status
Control
Persistent
AF Status
