# Supplementary figures and images for: Epigenetic Liquid Biopsy Marks Atrial Fibrillation: Evidence from the AF Big Picture Study
Source: Epigenomes. 2026 Feb 5;10(1):9. doi: 10.3390/epigenomes10010009 (PMC12922129; doi:10.3390/epigenomes10010009)

## Slide 1
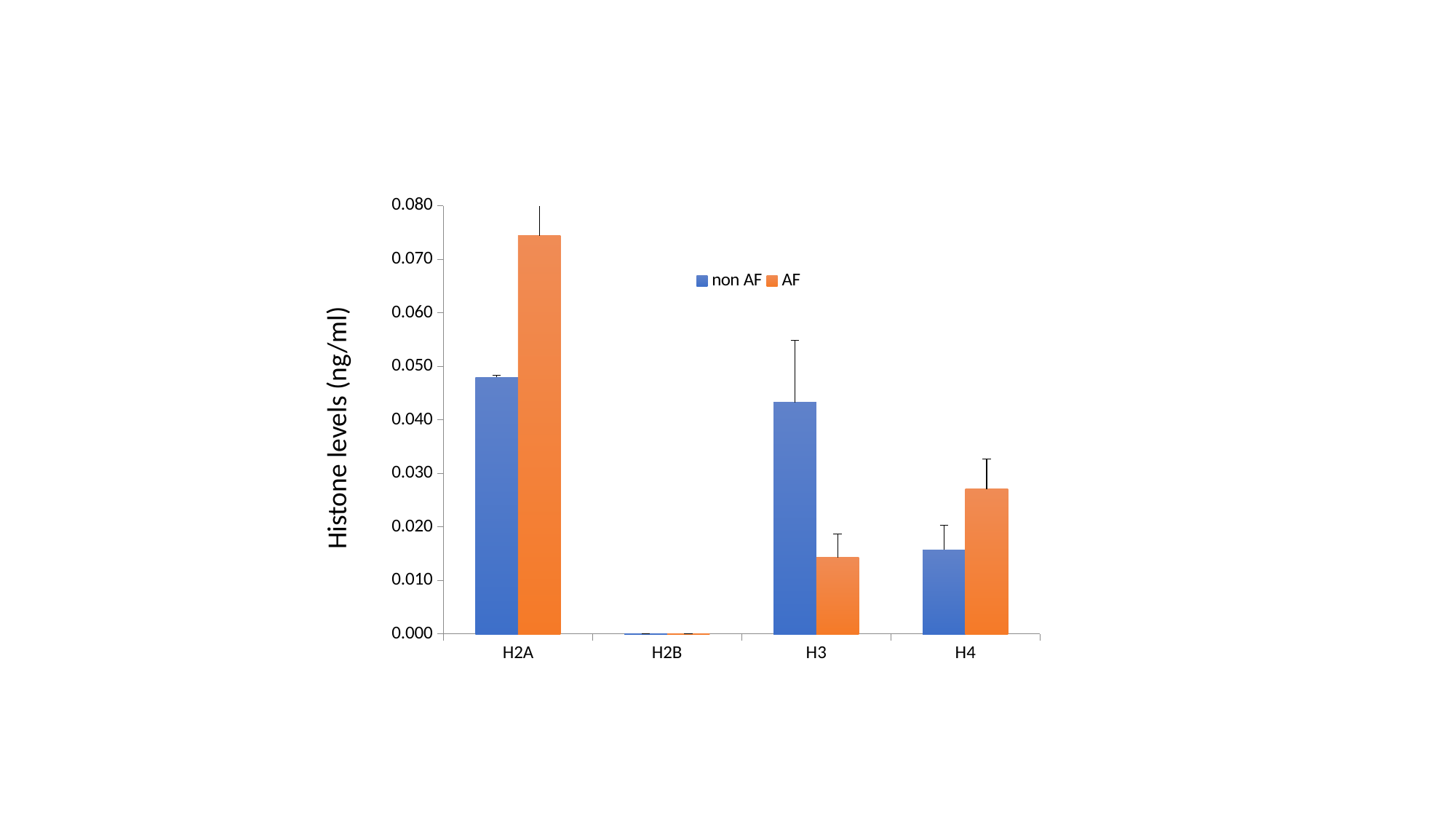

### Chart
| Category | non AF | AF |
|---|---|---|
| H2A | 0.04799250000000001 | 0.07441760138050042 |
| H2B | 0.0 | 0.0 |
| H3 | 0.043286219081272115 | 0.014310954063604261 |
| H4 | 0.01574930619796485 | 0.027081406105457912 |Histone levels (ng/ml)

Supplement: Supplementary file 1 [file epigenomes-10-00009-s001.zip › Supplemental Figure 1.pptx]
